# Corrigendum to: Vitamin B6 prevents Isocarbophos-induced posterior cerebral artery injury in offspring rats through up-regulating S1P receptor expression: Corrigendum

**DOI:** 10.3724/abbs.2025088

**Published:** 2025-08-05

**Authors:** Yanhua Liu, Kunli Yang, Ling Wang, Jinfang Yang, Yang Wang, Hu Luo, Peng Li, Yaling Yin

*Acta Biochim Biophys Sin*2021, 53(12): 1691–1701

https://doi.org/10.1093/abbs/gmab150

In the original version of this manuscript, an error was found in Figure 2B(Vit B6 + Fingolimod), Figure 5(Saline) and Figure 7 (Isocarbophos/Control) respectively. The correct figures are shown as follows. The authors apologize for the error. The corrigendum does not affect the interpretation odata and conclusions.

**Figure:**
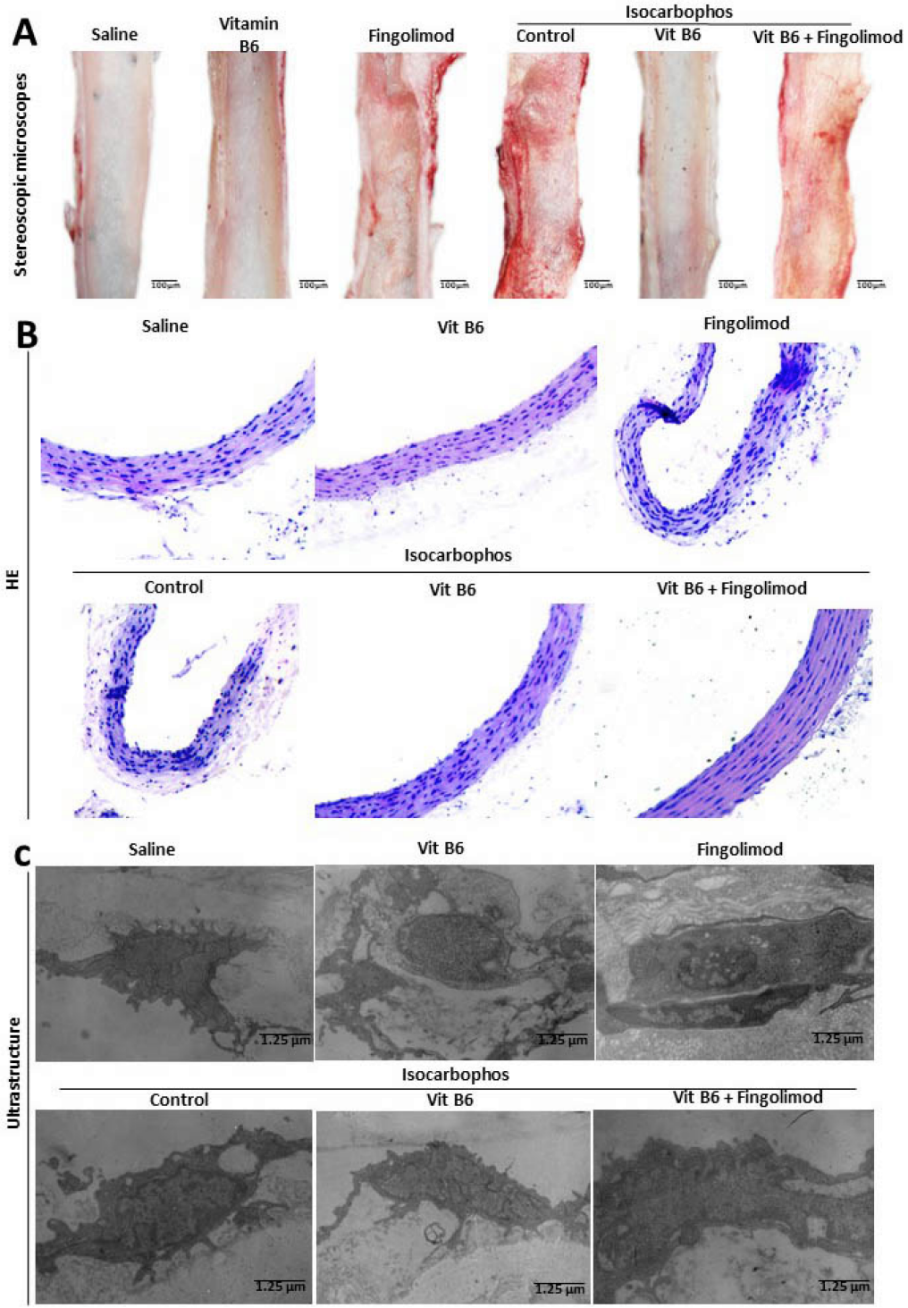
Figure 2 Injury analysis of posterior cerebral artery (A) Optical images of endothelial structure of posterior cerebral artery in offspring rats. (B) H&E staining images of posterior cerebral artery in offspring rats after different treatments. (C) Electron microscopy images of vascular endothelium of posterior cerebral artery in offspring rats.

**Figure:**
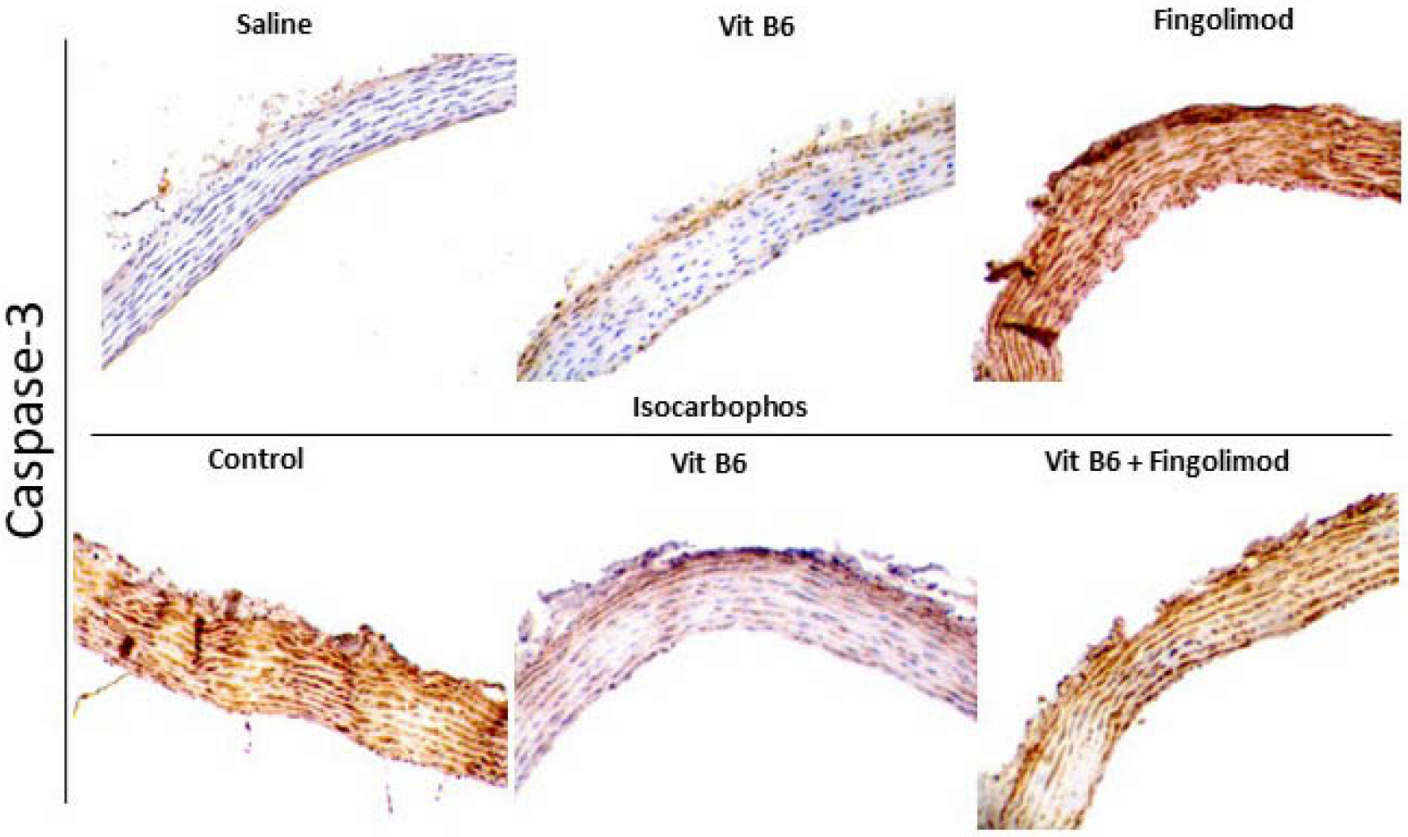
Figure 5 Expression of Caspase-3 in the posterior cerebral arteries of offspring rats in different treatment groups

**Figure:**
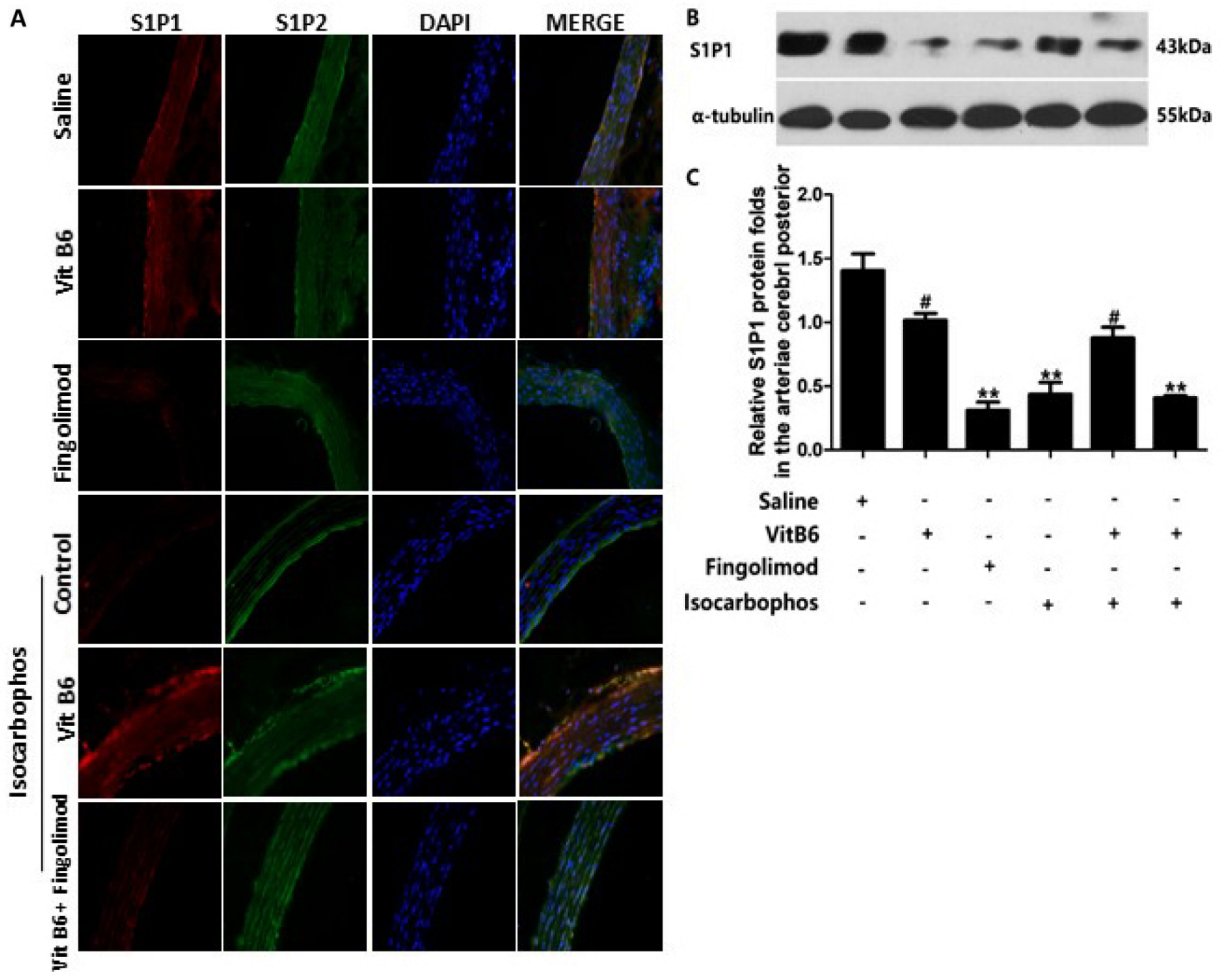
Figure 7 S1P receptor expression in different treatment groups (A) Immunofluorescence staining images of S1P1 and S1P2 receptor expression in the vascular tissues of offspring rats after different treatments. Red fluorescence signals indicate the S1P1 receptor expression, green fluorescence signals indicate the S1P2 receptor expression, and blue fluorescence signals are the cell nuclei stained by DAPI. (B) Expression of S1P1 receptor determined by western blot analysis. (C) Quantitative analysis of the S1P1 receptor expression in (B). #P < 0.05 vs Saline group; **P < 0.01 vs Isocarbophos group.

